# Beta to theta power ratio in EEG periodic components as a potential biomarker in mild cognitive impairment and Alzheimer’s dementia

**DOI:** 10.1186/s13195-023-01280-z

**Published:** 2023-08-07

**Authors:** Hamed Azami, Christoph Zrenner, Heather Brooks, Reza Zomorrodi, Daniel M. Blumberger, Corinne E. Fischer, Alastair Flint, Nathan Herrmann, Sanjeev Kumar, Krista Lanctôt, Linda Mah, Benoit H. Mulsant, Bruce G. Pollock, Tarek K. Rajji, on behalf of the PACt-MD Study Group

**Affiliations:** 1 Campbell Family Mental Health Research Institute, Centre for Addiction and Mental Health, 80 Workman Way, Toronto, ON M6J 1H4 Canada; 2 Adult Neurodevelopment and Geriatric Psychiatry Division, Centre for Addiction and Mental Health, Toronto, ON Canada; 3 Department of Psychiatry, Temerty Faculty of Medicine, University of Toronto, Toronto, ON Canada; 4 Temerty Centre for Therapeutic Brain Intervention, Centre for Addiction and Mental Health, Toronto, ON Canada; 5 Keenan Research Centre for Biomedical Science, St. Michael’s Hospital, Toronto, ON Canada; 6 University Health Network, Toronto, ON Canada; 7 Sunnybrook Health Sciences Centre, Toronto, ON Canada; 8 Rotman Research Institute, Baycrest Health Sciences Centre, Toronto, ON Canada; 9 Toronto Dementia Research Alliance, University of Toronto, Toronto, ON Canada

**Keywords:** Alzheimer’s dementia, Mild cognitive impairment, EEG, Periodic EEG power components

## Abstract

**Background:**

Alzheimer’s dementia (AD) is associated with electroencephalography (EEG) abnormalities including in the power ratio of beta to theta frequencies. EEG studies in mild cognitive impairment (MCI) have been less consistent in identifying such abnormalities. One potential reason is not excluding the EEG aperiodic components, which are less associated with cognition than the periodic components. Here, we investigate whether aperiodic and periodic EEG components are disrupted differently in AD or MCI vs. healthy control (HC) individuals and whether a periodic based beta/theta ratio differentiates better MCI from AD and HC groups than a ratio based on the full spectrum.

**Methods:**

Data were collected from 44 HC (mean age (SD) = 69.1 (5.3)), 114 MCI (mean age (SD) = 72.2 (7.5)), and 41 AD (mean age (SD) = 75.7 (6.5)) participants. Aperiodic and periodic components and full spectrum EEG were compared among the three groups. Receiver operating characteristic curves obtained via logistic regression classifications were used to distinguish the groups. Last, we explored the relationships between cognitive performance and the beta/theta ratios based on the full or periodic spectrum.

**Results:**

Aperiodic EEG components did not differ among the three groups. In contrast, AD participants showed an increase in full spectrum and periodic relative powers for delta, theta, and gamma and a decrease for beta when compared to HC or MCI participants. As predicted, MCI group differed from HC participants on the periodic based beta/theta ratio (Bonferroni corrected* p*-value = 0.036) measured over the occipital region. Classifiers based on beta/theta power ratio in EEG periodic components distinguished AD from HC and MCI participants, and outperformed classifiers based on beta/theta power ratio in full spectrum EEG. Beta/theta ratios were comparable in their association with cognition.

**Conclusions:**

In contrast to a full spectrum EEG analysis, a periodic-based analysis shows that MCI individuals are different on beta/theta ratio when compared to healthy individuals. Focusing on periodic components in EEG studies with or without other biological markers of neurodegenerative diseases could result in more reliable findings to separate MCI from healthy aging, which would be valuable for designing preventative interventions.

**Supplementary Information:**

The online version contains supplementary material available at 10.1186/s13195-023-01280-z.

## Background

Alzheimer’s dementia (AD) is a progressive illness that accounts for 60–80% of all dementia [1]. AD is typically preceded by mild cognitive impairment (MCI) during which individuals can still function independently [2]. Thus, biological markers that distinguish MCI from AD or healthy aging are crucial to the development of preventative interventions which in turn could improve quality of life, caregiver burden, and cost of care due to dementia [3]. Of note that while MCI is often associated with an increased risk of AD, it does not inevitably lead to its development [2].

Electroencephalography (EEG) is a non-invasive and inexpensive tool that allows the assessment of neural ionic current flows based on differences in voltages at different spatial scales in the brain and at a high time resolution [4–6]. Most EEG, like magnetoencephalogram (MEG), studies in AD and MCI have analyzed the power spectral density (PSD), especially for resting-state EEG [5, 7–12]. In general, these studies have found increased power in delta and theta and decreased powers in alpha and beta in individuals with AD, especially in the temporal and posterior/occipital brain regions [5, 7–14]. The ratios of fast-to-slow frequency powers have also been shown to differ between individuals with AD and those with normal cognition [3]. One of the most promising power ratios is the ratio of fast beta frequency to slow theta frequency (“beta/theta ratio”), suggesting that the beta/theta ratio is a marker of cognitive processing capacity [4–8].

In contrast, EEG differences in MCI vs. healthy individuals have been smaller in magnitude and not consistently replicated [9, 15–17], including when measured over the occipital or temporal regions [9, 18]. In fact, the EEG power spectrum-based parameters are more sensitive to identify the MCI subjects most likely to progress to dementia in comparison with those MCI subjects that do not develop [15]. One potential reason is that these studies did not exclude the aperiodic component of EEG. EEG power spectra typically consist of two main components: an aperiodic background part of the spectrum (arrhythmic component - 1/f-like component); and periodic or rhythmic neural oscillations [19]. The aperiodic part of EEG is also called fractal or “scale-free” activity because the signal of the aperiodic part is typically self-similar across many temporal scales [20]. Several studies have shown the benefit of focusing on the periodic parts of EEGs [15, 19, 21, 22]. The periodic components of EEG, rather than the aperiodic components, have been associated with speed of processing and working memory [15]. Excluding the aperiodic parts of an EEG could be beneficial because the aperiodic parts could mask observing reductions in true oscillatory power, shifts in oscillation center frequency, or reductions in broadband power [19], all of which can be subtle in mild disease conditions such as MCI.

To our knowledge, the effect of excluding the aperiodic component of EEG has not been studied in AD and MCI. In this context, we conducted a study to investigate whether we could better distinguish MCI from healthy control (HC) and AD participants using periodic only vs. full spectrum analyses of eyes-closed resting-state EEG, with a focus on fast-to-slow activity as measured using beta-to-theta power ratio.

## Material and methods

### Participants

HC and MCI participants were recruited for an AD prevention trial (Prevention of AD with Cognitive Remediation plus transcranial Direct Current Stimulation in Mild Cognitive Impairment and Depression (PACt-MD); NCT02386670) across five academic hospitals in Toronto, Canada. The complete clinical trial design and rationale have been reported previously [16]. All participants provided written informed consent as approved by the local Research Ethics Board and Clinical Trials Ontario. Eligibility criteria for all participants included: (1) no lifetime Diagnostic and Statistical Manual of Mental Disorders Fifth Edition (DSM 5) [17] diagnosis of schizophrenia, bipolar disorder, or obsessive-compulsive disorder; (2) no significant neurological conditions impacting cognition or unstable medical illnesses; (3) no DSM 5 diagnosis of alcohol or other substance use disorder within the past 12 months; (4) no use of cognitive enhancers in the 6 weeks prior to entering the study; and (5) Montgomery–Asberg Depression Rating Scale [18] (MADRS) score ≤ 10. Additional eligibility criteria for the MCI group included: (1) Age ≥ 60; and (2) DSM-5 diagnosis of Mild Neurocognitive Disorder. Additional eligibility criteria for the HC group included: (1) Age ≥ 60; (2) no DSM 5 diagnosis of Mild or Major Neurocognitive Disorder; and (3) no neuropsychological testing done in the 12 months prior to baseline assessment. In addition, MCI diagnosis was confirmed at a consensus conference that included the clinical psychiatrist of the participant, one or two principal investigators, the study neuropsychologist, and the research staff who administered the clinical, functional, and neuropsychological assessments. The EEG study was an optional portion of the parent study. Of those who consented and completed the EEG at baseline, 114 MCI and 44 HC participants were included in the final analyses (see CONSORT chart, Figs. S1 and S2 in Supplementary Appendix A). Of note, PACt-MD also recruited participants with a major depressive disorder with or without MCI. These participants were not included in this analysis.

AD participants were included from two other intervention studies (clinicaltrials.gov identifiers: NCT01847586 and NCT02537496). Eligibility criteria for AD participants included: (1) a diagnosis of probable AD according to the criteria of either the National Institute of Neurological and Communicative Disorders and Stroke and the Alzheimer’s Disease and Related Disorders Association (NINCDS-ADRDA) [23] in one study or the National Institute on Aging – Alzheimer’s Association (NIA-AA) Research Framework for Alzheimer’s Disease [24] in the other study; (2) either not taking an acetylcholinesterase inhibitor or having been on a stable dosage for at least 3 months; and (3) no DSM-5 diagnosis other than Major Neurocognitive Impairment due to Alzheimer’s disease (i.e., AD) within the past 12 months. In addition, in one study, participants were 65 years old or older and they had a Mini Mental State Examination (MMSE) [25] score ≥ 17; in the other study, they were 55 years old or older and they had a Montreal Cognitive Assessment (MoCA) [26] score ≥ 10 (see CONSORT chart, Fig. S3 in Supplementary Appendix).

All participants in the three studies provided written informed consent, as approved by the Research Ethics Board at the Centre for Addiction and Mental Health, Toronto, Canada.

### Cognitive assessments

#### Clinical

The MCI and HC participants completed both the MoCA and MMSE. AD participants did not undergo both tests: 26 completed the MMSE and 14 completed the MoCA. For one participant, neither a MMSE nor a MoCA score was available. For this analysis, for the 26 participants who had completed the MMSE, we generated and used equivalent MoCA scores based on the conversion table from [27].

### EEG data collection and processing

All three studies were conducted using the same EEG equipment and protocols as described in [17]. Briefly, EEGs were completed using a 64-channel Synamps 2 EEG device and the 10–10 montage system. Electrodes were referenced to CPz. EEG signals were recorded for 10 min at the sampling frequency of 1000 Hz. Participants sat on a chair, eyes closed in a relaxed state while avoiding to move their head or eyes, or to sleep. A band-pass filter with cut-off frequencies of 1 and 45 Hz was next used, bad channels were removed, and the data was re-referenced to a common average reference.

EEG data processing occurred offline using MATLAB (The MathWorks, Inc.) and EEGLAB toolbox. Initially, we visually inspected the EEG data to ensure the absence of prominent delta and theta waves, which are typically indicative of sleep EEG patterns, in order to exclude drowsiness during the EEG recording. Subsequently, we conducted a visual examination of the EEG data to eliminate segments with noticeable noise and channels heavily affected by various artifacts, including motion artifacts, eye movements, and blinks. Furthermore, in an effort to further mitigate any remaining noise, we divided the processed continuous EEG data into epochs of 2 s each and applied independent component analysis (ICA) to remove components related to eye movements and muscle activity [28].

After the pre-processing step, we calculated the total power spectrum using the Welch method [29] with a Hann window function and a segment size 2 s with overlap of 50%, and taking the median. Subsequently, we used the “fooof” (fitting of one over f) toolbox to parametrize the resulting total power spectrum; it performs a sequential decomposition of the power spectrum into its aperiodic and periodic components, optimizing the modelled spectrum using a least-squared- error approach [19]. We then normalized the resulting periodic component of the spectrum (by total power of the periodic component) to account for inter-individual differences when computing averages.

We calculated the total PSD of EEG data based on the Welch method [29] in the frequency domain 1–45 Hz, which includes delta (1–4 Hz), theta (4–8 Hz), alpha (8–13 Hz), beta (13–30 Hz), and gamma (30–45 Hz). The algorithm considers the power spectrum as a combination of an aperiodic background component with overlying periodic components, or oscillations. These assumed periodic oscillatory power components are characterized as frequency regions of power above the aperiodic or background component, and are referred to here as ‘peaks’. The algorithm operates on PSDs in semilog-power space, where the frequencies are linearly spaced, and the power values are log-spaced. The aperiodic component is fitted as a function over the entire range of the spectrum, while each oscillatory peak is modeled using a Gaussian. Each Gaussian is an oscillation, and the three parameters that define it are used to describe the oscillation. This formulation models the power spectrum as:

$$PSD=L+\sum_{n=0}^{N}{G}_{n},$$

where the PSD is comprised of two parts: the aperiodic component, *L*, and *N* total Gaussians, *G*. Each ${G}_{n}$ is a Gaussian fitted to a peak, with *N* representing the total number of peaks extracted from the power spectrum as:

$${G}_{n}=a\times exp\left(\frac{-{(F-c)}^{2}}{{2w}^{2}}\right),$$

where *a* denotes the power of the peak, in log10(power) values; *c* shows the center frequency in Hz; *w* is the standard deviation of the Gaussian in Hz; and *F* is the vector of input frequencies. The aperiodic or background component, *L*, is modeled based on a Lorentzian function as follows:

$$L=b-log\left(K+{F}^{x}\right),$$

where *b* is the broadband offset, χ is the exponent and *k* is the ‘knee’ parameter that controls the bend in the aperiodic component, *F* denotes the vector of input frequencies. If *k* equals 0, this formulation is equivalent to fitting a line in log–log space, which is also known as fixed mode. It is worth mentioning that there is a direct relationship between the slope, *a*, of the line in log–log spacing, and the exponent, χ, with χ equaling negative *a* when there is no knee. By fitting with *k*, it is possible to parameterize bends or knees in the aperiodic component that occur across broad frequency ranges, which is particularly relevant in intracranial recordings [19].

We utilized the parameter settings for the fooof algorithm (peak width limits = [1,13], maximum number of peaks = 3, minimum peak height = 0.00, peak threshold = 1.5, and aperiodic mode = ‘fixed’) [19, 30]. We validated visually that these settings achieved an adequate fit and that there was no distinct ‘knee’ observed within the 1–45 Hz frequency range we analyzed.

### Statistical analyses

All data were analyzed using the Statistical Program for Social Sciences (SPSS) version 23.0 (SPSS Inc., Chicago, IL, USA). One-way analysis of variance (ANOVA) and *χ*^2^ tests were used to evaluate differences among the three groups on demographic, clinical and MoCA measures. The level of significance was set at *α* = 0.05. If needed, data were transformed using natural log (LN) to approximate the normal distributional assumptions required by parametric statistical methods. Since there were group differences in age and education, these variables were included as covariates in subsequent analyses.

As Alzheimer’s disease can affect whole the brain and due to the effect of volume conduction in electrode-based EEG analysis, we investigate the effect of AD and MCI on both the global and local brain activity patterns. We first generated full spectrum, periodic, aperiodic spectra for all frequencies across the whole brain (averaged across all EEG electrodes) in AD, HC, and MCI participants. We compared the three groups on relative powers across all frequencies for full or periodic spectra analyses in five brain regions, including frontal (AF3, AF4, FP1, FPZ, FP2, F7, F5, F3, F1, FZ, F2, F4, F6, F8, FT7, FC5, FC3, FC1, FCZ, FC2, FC4, FC6, FT8), temporal (T7, T8, TP7, TP8), central (C5, C3, C1, CPz, C2, C4, C6, CP5, CP3, CP1, CPZ, CP2, CP4, CP6), parietal (P7, P5, P3, P1, Pz, P2, P4, P6, P8 PO7, PO5, PO3, POz, PO4, PO6, PO8), and occipital (O1, O2, Oz).

Then, we generated the beta/theta ratios using full or periodic spectra and compared them in the three groups. All group analyses were conducted using Analysis of Covariance (ANCOVA) with the EEG measure as the dependent variable, group as the independent variable and age and education as the covariates. Each ANCOVA was followed by *post-hoc* analyses with Bonferroni correction for 90 comparisons (3 (HC vs. MCI, HC vs. AD, and MCI vs AD) * 5 (number of brain regions) * 6 (delta, theta, alpha, beta, gamma, and beta/theta power ratio)).

We also conducted a post-hoc power analysis to determine the minimum effect sizes that our sample size had sufficient power to detect. By providing this information, we better evaluated the reliability of our findings and determine if the observed effect sizes were large enough to be detected with the sample size employed.

Then, given our primary aim, we applied a logistic regression to distinguish HC from MCI and AD participants, and MCI from AD participants, using beta/theta ratios in the occipital lobe. We used a 10-fold cross validation (CV) and evaluated the cross-validated performance of each model using the area under the Receiver Operating Characteristic curve (AUC_ROC_). To improve the estimated performance of the models, we repeated the 10-fold CV process 10 times and reported the average outcomes. To interpret the classification findings, we generated ROC curves using the MoCA given that the MoCA is often used as a screening tool for AD and MCI. These analyses incorporated age and education as covariates to account for the observed group differences in these variables. Both variables were included as covariates alongside the independent (predictor) variable (beta/theta ratio for the periodic components of EEG, beta/theta ratio for the full power spectrum, or MoCA score). This allowed us to control for the potential influence of age and education on the relationship between each predictor variable and the outcome variable (HC, MCI, and AD groups).

We also calculated the DeLong test *p*-value to evaluate whether the AUCs from the two models (beta/theta ratios for the periodic components of EEG vs. full power spectrum in the occipital lobe) are statistically significantly different [31].

Last, we explored the relationships between cognitive performance across all groups based on MoCA scores and the beta/theta ratios in the occipital lobe calculated based on the full spectrum or periodic spectrum. To this end, we conducted a linear regression to assess the association between beta/theta ratio as the independent variable and MoCA score as the dependent variable while controlling for group, age and education as covariates given that the groups differed in age and education.

## Results

All participants demographic, clinical and cognitive characteristics are presented in Table 1. The groups differed in age, education, and MoCA scores.

**Table 1:** Demographic, clinical, and cognitive characteristics

	**HC**	**MCI**	**AD**	***F* or χ2 (df1, df2)**	***P* values**
*N*	44	114	41		-
*Age in years (SD)*	69.07 (5.31)	72.19 (7.45)	75.65 (6.53)	9.81 (2, 196)	< 0.001 HC vs MCI, *p* = 3e-03; HC vs AD *p* = 3e-05; MCI vs AD, *p* = 3e-03
*Gender (F:M)*	31: 13	72: 42	24: 17	0.67 (2, 196)	NS
*Highest level of education*				16.5 (2, 196)	2e-07 HC vs MCI, *p* = 0.28; HC vs AD *p* = 2e-07; MCI vs AD, *p* = 2e-06
*Less than high school*	1	6	10		
*High school graduate*	2	10	8		
*Partial University*	2	9	7		
*University degree*	26	65	10		
*Graduate degree*	13	24	6		
*MoCA score (SD)*	27.65 (1.31)	23.75 (2.46)	17.17 (3.93)	168.61 (2, 196)	1e-44; HC vs MCI, *p* = 2e-24; HC vs AD *p* = 4e-45; MCI vs AD, *p* = 7e-32

### Whole brain full power spectrum EEG vs. periodic and aperiodic components

**Fig. 1:**
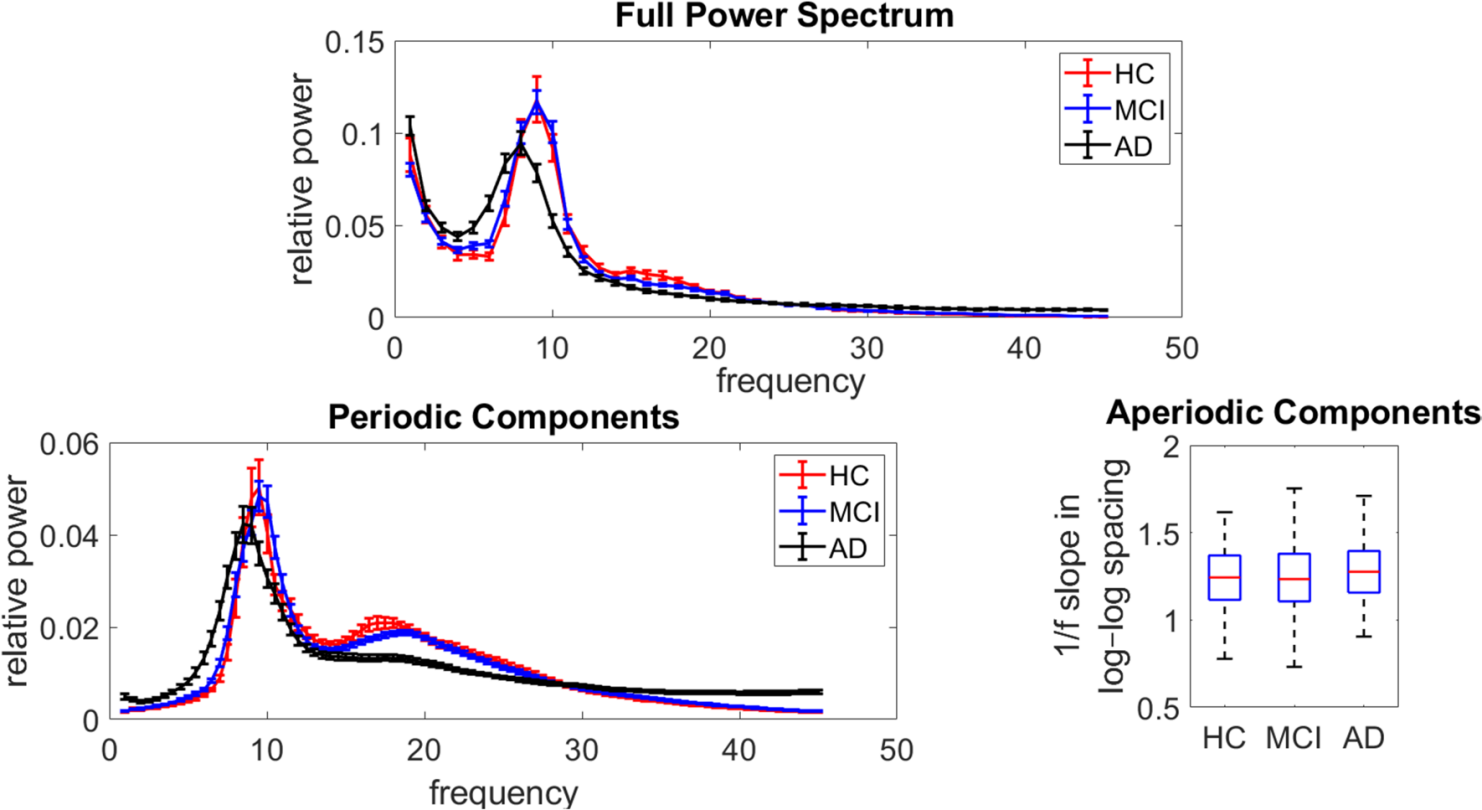
Whole-brain averaged relative power spectral density curves for full power spectrum and periodic components (rhythmic oscillations) in addition to fractal background components (aperiodic part or 1/f-like noise in log-log spacing) in Healthy Control (HC) (red), Mild Cognitive Impairment (MCI) (blue), and Alzheimer’s dementia (AD) (black) participants. In the full power and periodic components panels error bars represent standard errors

The full power spectrum and its periodic and aperiodic background components are shown in Fig. 1, averaged across all electrodes. The figure shows a qualitative increase of relative power for delta, theta, and gamma, and a decrease of relative power for beta using the full spectrum and the periodic component in AD participants compared to HC and MCI participants (we quantitatively compared these metrics for different regions in the next subsection). In contrast, there are no differences in aperiodic background EEG components among HC, MCI, and AD participants (ANCOVA *F*(3,195) = 0.55, *p* = 0.56).

### Full spectrum vs. periodic EEG relative powers in different brain regions

**Fig. 2:**
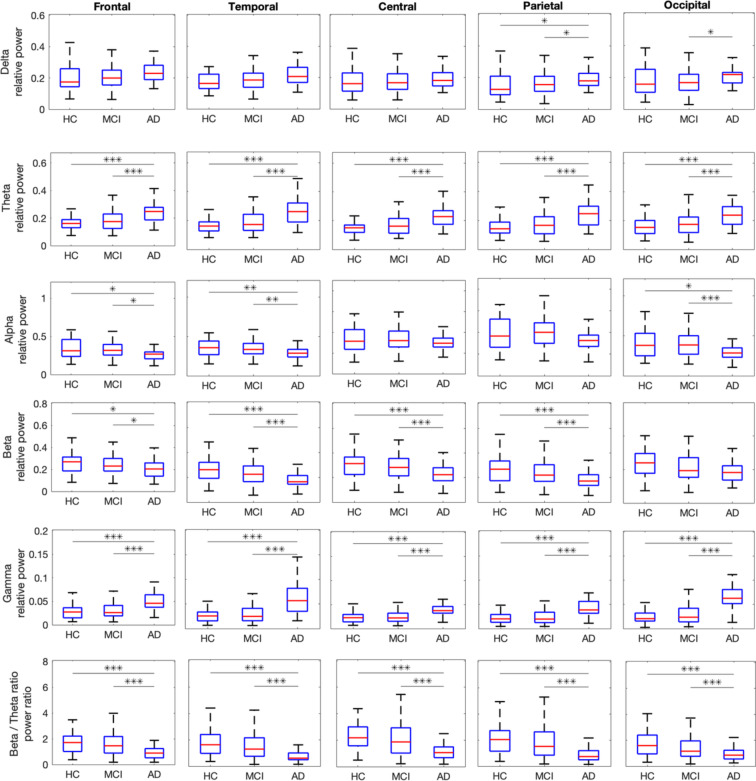
Evaluation of regional full power spectrum in delta, theta, alpha, beta, gamma, and beta/theta power ratio on the frontal, occipital, central, parietal, and occipital lobes for HC, MCI, and AD. Bonferroni corrected post-hoc comparisons with *p*-values smaller than 0.05, 0.01, and 0.001 are shown with *, **, and ***, respectively

**Fig. 3:**
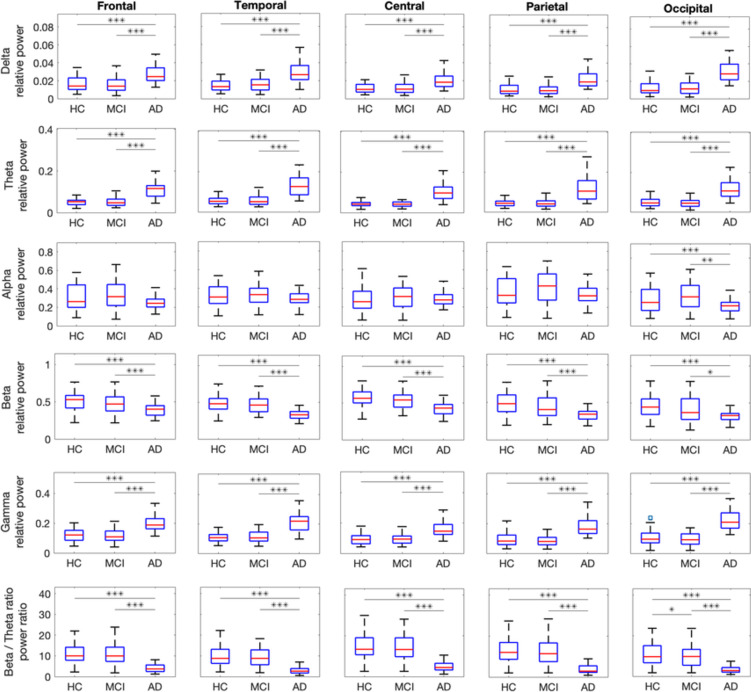
Evaluation of regional EEG periodic components in delta, theta, alpha, beta, gamma, and beta/theta power ratio on the frontal, occipital, central, parietal, and occipital lobes for HC, MCI, and AD. Bonferroni corrected post-hoc comparisons with *p*-values smaller than 0.05, 0.01, and 0.001 are shown with *, **, and ***, respectively

We next evaluated whether there were differences in the full power spectrum and periodic components in five main brain regions (frontal, temporal, central, parietal, and occipital). The relative powers of these full and periodic oscillations are shown in Figs. 2 and 3, respectively, for each group in delta, theta, alpha, beta, and gamma as well as beta/theta power ratio. The results of the ANCOVAs are shown in Table 2.

**Table 2:** Full spectrum vs. periodic EEG relative powers in different brain regions

	Full Power Spectrum (All Oscillations)			Periodic Components or Oscillations		
	*F*	ANCOVA *p*-value (post-hoc *p*-values HC vs. MCI, HC vs. AD, MCI vs. AD)	Observed power	*F*	ANCOVA *p*-value (post-hoc p-values HC vs. MCI, HC vs. AD, MCI vs. AD)	Observed power
Delta in frontal region	2.510	0.060	0.62	14.516	< 0.001 (1.000, < 0.001, < 0.001)	1
Theta in frontal region	10.974	< 0.001 (0.660, < 0.001, < 0.001)	1	22.396	< 0.001 (0.252, < 0.001, < 0.001)	1
Alpha in frontal region	3.369	0.020 (1.000, 0.041, 0.012)	0.76	0.896	0.444	0.24
Beta in frontal region	3.233	0.023 (0.660, 0.015, 0.033)	0.74	7.961	< 0.001 (0.786, < 0.001, < 0.001)	0.99
Gamma in frontal region	13.457	< 0.001 (1.000, < 0.001, < 0.001)	1	21.086	< 0.001 (0.541, < 0.001, < 0.001)	1
FSA ratio in frontal region	7.839	< 0.001 (0.713, < 0.001, < 0.001)	0.99	23.918	< 0.001 (0.219, < 0.001, < 0.001)	1
Delta in temporal region	2.720	0.046 (1.000, 0.077, 0.110)	0.66	18.053	< 0.001 (1.000, < 0.001, < 0.001)	1
Theta in temporal region	12.252	< 0.001 (0.366, < 0.001, < 0.001)	1	24.591	< 0.001 (0.192, < 0.001, < 0.001)	1
Alpha in temporal region	5.322	0.002 (1.000, 0.004, 0.002)	0.93	1.275	0.284	0.34
Beta in temporal region	7.620	< 0.001 (0.421, < 0.001, < 0.001)	0.99	26.335	< 0.001 (0.335, < 0.001, < 0.001)	1
Gamma in temporal region	16.176	< 0.001 (1.000, < 0.001, < 0.001)	1	28.305	< 0.001 (0.459, < 0.001, < 0.001)	1
FSA ratio in temporal region	10.659	< 0.001 (0.339, < 0.001, < 0.001)	1	30.223	< 0.001 (0.164, < 0.001, < 0.001)	1
Delta in central region	1.315	0.271	0.35	12.863	< 0.001 (1.000, < 0.001, < 0.001)	1
Theta in central region	13.352	< 0.001 (0.892, < 0.001, < 0.001)	1	26.082	< 0.001 (0.483, < 0.001, < 0.001)	1
Alpha in central region	0.477	0.698	0.15	0.511	0.675	0.15
Beta in central region	7.139	< 0.001 (0.721, < 0.001, 0.001)	0.98	16.331	< 0.001 (0.699, < 0.001, < 0.001)	1
Gamma in central region	11.950	< 0.001 (1.000, < 0.001, < 0.001)	1	18.738	< 0.001 (0.664, < 0.001, < 0.001)	1
FSA ratio in central region	11.440	< 0.001 (0.705, < 0.001, < 0.001)	1	28.458	< 0.001 (0.429, < 0.001, < 0.001)	1
Delta in parietal region	3.368	0.020 (1.000, 0.032, 0.041)	0.78	18.110	< 0.001 (1.000, < 0.001, < 0.001)	1
Theta in parietal region	14.218	< 0.001 (0.859, < 0.001, < 0.001)	1	25.526	< 0.001 (0.463, < 0.001, < 0.001)	1
Alpha in parietal region	2.560	0.056	0.62	0.540	0.656	0.16
Beta in parietal region	6.323	< 0.001 (0.502, < 0.001, < 0.001)	0.97	11.123	< 0.001 (0.427, < 0.001, < 0.001)	1
Gamma in parietal region	15.532	< 0.001 (1.000, < 0.001, < 0.001)	1	23.880	< 0.001 (1.000, < 0.001, < 0.001)	1
FSA ratio in parietal region	12.331	< 0.001 (0.520, < 0.001, < 0.001)	1	30.233	< 0.001 (0.280, < 0.001, < 0.001)	1
Delta in occipital region	3.785	0.011 (1.000, 0.142, 0.019)	0.81	23.919	< 0.001 (1.000, < 0.001, < 0.001)	1
Theta in occipital region	10.142	< 0.001 (0.482, < 0.001, < 0.001)	1	22.913	< 0.001 (0.179, < 0.001, < 0.001)	1
Alpha in occipital region	6.670	< 0.001 (1.000, 0.014, < 0.001)	0.97	3.978	0.009 (0.878, 0.239, 0.005)	0.83
Beta in occipital region	1.878	0.135	0.48	6.529	< 0.001 (0.165, 0.001, 0.021)	0.97
Gamma in occipital region	25.417	< 0.001 (1.000, < 0.001, < 0.001)	1	27.331	< 0.001 (1.000, < 0.001, < 0.001)	1
FSA ratio in occipital region	7.011	< 0.001 (0.283, < 0.001, 0.001)	0.98	28.456	< 0.001 (0.036, < 0.001, < 0.001)	1

The post-hoc comparisons were done when ANCOVA *p*-values < 0.05. The degrees of freedom for all the tests between-subjects effects were 3

Table 2 shows that while there was a group effect for almost all ANCOVAs. Bonferroni-corrected post-hoc analyses revealed a significant difference between MCI and HC participants specifically in the periodic spectrum based on the occipital lobe beta/theta ratio (*F*(3; 195) = 28.456, *p* < 0.001) with the ratio in MCI being lower than in HC participants (*p* = 0.036; Cohen’s *d* = 0.52). The observed power of 1 for the periodic spectrum based the occipital lobe beta/theta ratio suggests that the sample size and the effect size in the study were sufficient to detect the expected effect with maximum certainty.

### Classification analysis

**Fig. 4:**
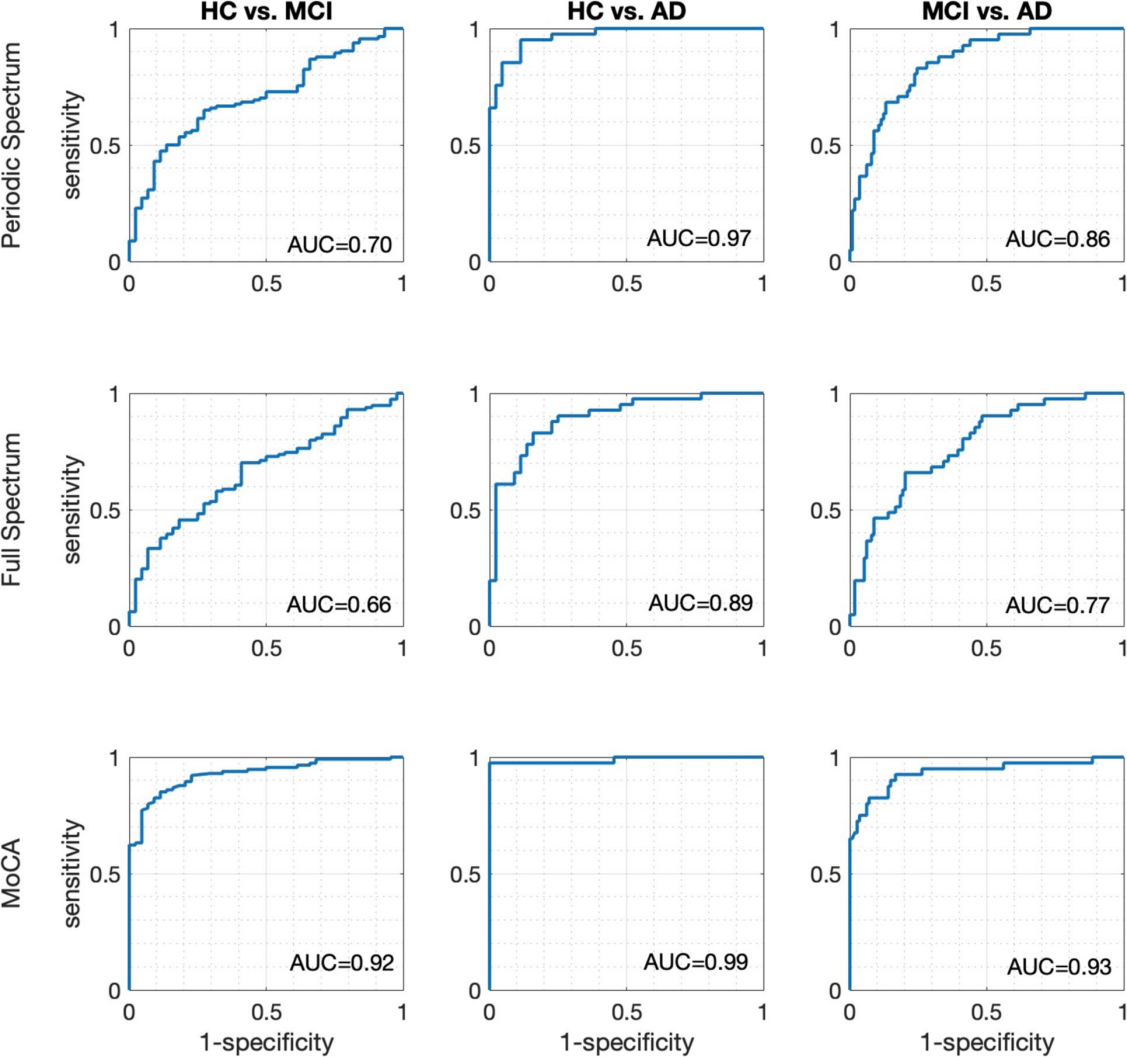
Classification performance of beta/theta power ratios for periodic- and full spectrum-based analyses, and MoCA total scores in discriminating AD, MCI, and HC participants. Both beta/theta power ratios were calculated from the occipital region. To address the influence of group differences in age and education, these variables were taken into account as covariates in these analyses

Discriminating participants using the periodic spectrum-based occipital lobe beta/theta ratio was consistently better than using the full spectrum occipital lobe beta/theta ratio (Fig. 4; AD vs. HC: AUC_ROC_ = 0.97 vs. AUC_ROC_ = 0.89; AD vs. MCI: AUC_ROC_ = 0.86 vs. AUC_ROC_ = 0.77; and MCI vs. HC: AUC_ROC_ = 0.70 vs. AUC_ROC_ = 0.66). The AUC values obtained by the MoCA total scores (AD vs. HC: AUC_ROC_ = 0.99; AD vs. MCI: AUC_ROC_ = 0.93; and MCI vs. HC: AUC_ROC_ = 0.92) are higher than those for the periodic and full spectrum of EEG (Fig. 4). Based on the DeLong test, we found that the AUC differences between the periodic and full spectrum analyses were significant for HC vs. AD and MCI vs. AD (*p*’s < 0.05) but not for HC vs. MCI (*p* > 0.05).

### Associations between beta/theta ratio in occipital lobe and cognition

**Fig. 5:**
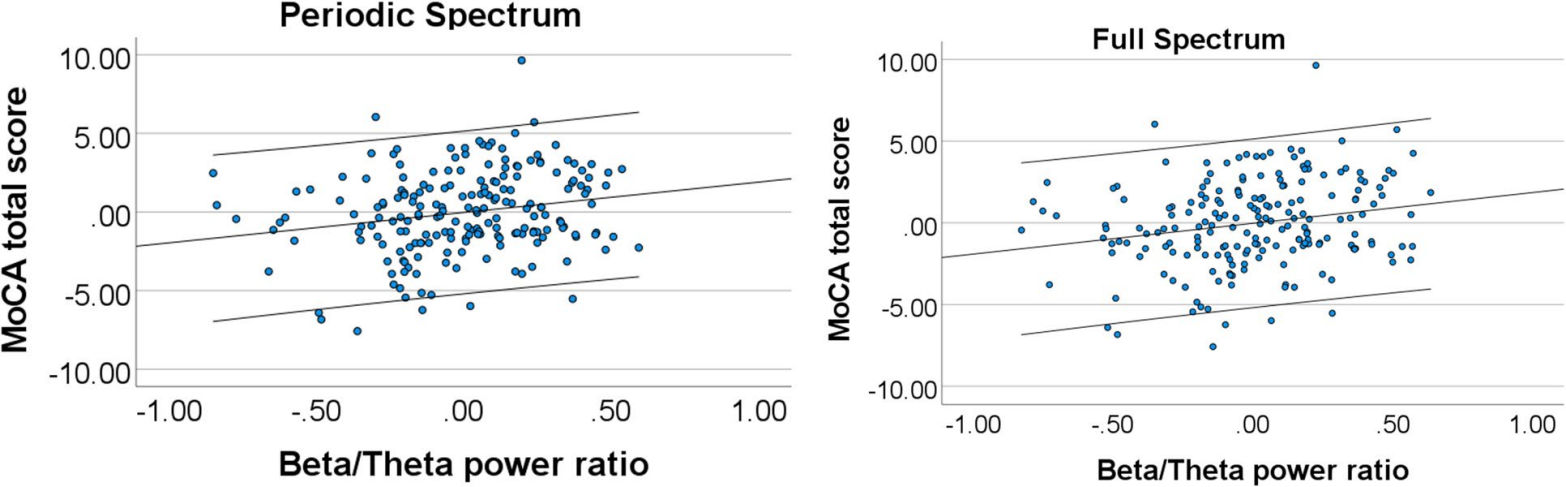
Partial regression plots demonstrating the associations between beta/theta power ratios and the Montreal Cognitive Assessment (MoCA) total score using periodic or full spectrum as captured in the occipital lobe for the Alzheimer’s dementia (AD), Mild Cognitive Impairment (MCI), and Healthy Control (HC) participants. The two parallel lines represent the 95% confidence intervals for each regression line

Given that the beta/theta ratio was different in MCI vs. HC participants only in the occipital lobe, we assessed the association with cognition using the occipital ratios (Fig. 5). Both linear regression models showed significant associations between beta/theta ratios and MoCA scores, after controlling for group, age and education: (periodic: *F*(4, 193) = 84.93, *p* < 0.001, *R* square = 0.638, beta coefficient for beta/theta ratio = 0.144, t-value = 2.804 and *p*-value = 0.006; vs. full spectrum: *F*(4, 193) = 86.08, *p* < 0.001, *R* square = 0.641, beta coefficient for beta/theta ratio = 0.140, t-value = 3.097 and *p*-value = 0.002). We also found that group was associated with MoCA score (periodic: beta coefficient = -0.663, t-value = -12.630 and *p*-value < 0.001; full spectrum: beta coefficient = -0.690, t-value = -14.012 and *p*-value < 0.001), but not age (periodic: beta coefficient = -0.054, t-value = -1.174 and *p*-value = 0.242; full spectrum: beta coefficient = -0.063, t-value = -1.385 and *p*-value = 0.168) and weakly with education (periodic: beta coefficient = 0.083, t-value = 1.783 and *p*-value = 0.076; full spectrum: beta coefficient = 0.094, t-value = 2.041 and *p*-value = 0.043).

## Discussion

In this study, we investigated whether resting-state EEG features based on periodic power components are better at distinguishing amongst AD, MCI and healthy individuals than EEG features based on full power spectrum, with a focus on MCI vs. healthy individuals. As expected, MCI were different than healthy individuals on the beta/theta ratio over the occipital lobe when calculated based on periodic power components and not the full spectrum. Otherwise, we found that full spectrum and periodic based features performed similarly. We also found that the logistic regression classifiers that used beta/theta power-periodic as an input feature are noticeably better in distinguishing AD vs. HC, and AD vs. MCI, than classifiers that used beta/theta power using full spectrum. Further, we found that the associations between performances on cognitive tasks and beta/theta power ratio were comparable between periodic components vs. full spectrum analysis. Last, we found no significant differences in the aperiodic components of EEGs among all groups.

Increases in powers of slow oscillations, especially theta oscillations, have been associated with AD and cerebrospinal fluid total tau accumulation [9, 10]. In contrast, increases in powers of fast oscillations, e.g. beta oscillations, are associated with better cognitive control, including attentional inhibition, cognitive set-maintenance and cognitive effort [11]. Thus, a fast-to-slow activity ratio like the beta/theta ratio would be expected to be a sensitive measure in separating individuals across a spectrum of cognitive decline given that a neurodegenerative process could be leading to increases in theta powers and decrease beta powers simultaneously. It was also shown that the ratio is a marker of cognitive processing capacity [4–8]. Additionally, there has been a longstanding hypothesis that the beta/theta power ratio is indicative of cognitive functioning, particularly in conditions like attention-deficit hyperactivity disorder, and executive function deficits [5, 7, 11–13]. Given that both executive function and cognitive processing capacity are impaired in MCI and AD, it is reasonable to anticipate a reduced beta/theta ratio in individuals with MCI when compared to healthy controls. The results are consistent with a recent study demonstrating the successful differentiation of individuals with MCI from HC using a similar spectral power ratio, specifically (alpha + beta)/(delta + theta) [32].

The aperiodic component of EEG may be related to excitation/inhibition balance with flatter slopes reflecting increased excitation and or decreased inhibition and so may be linked to aging [15, 19, 33]. This is agrees with the fact that increased age is associated with a flatter, less negatively sloped power spectrum [33]. This study showed that the EEG aperiodic power spectrum components may not be related to Alzheimer’s disease as a neurodegenerative disease. These results are also in agreement with [34] showing that the EEG spectral slowing in AD is driven by periodic components, while aperiodic EEG components remain unaffected. Apart from that, previous studies indicate that, although the periodic components of the EEG spectrum is linked to the processing speed the aperiodic component may not show a consistent relationship to cognitive performance [15].

The reason behind detecting a statistically significant difference between MCI and healthy individuals only over the occipital lobe could be because the occipital lobe is typically the least affected region by EEG main artifacts (e.g., eye and body movement, and electrocardiogram) [35]. This occipital lobe specificity also agrees with the literature on EEG features in AD or MCI but based on full power spectrum [13, 14].

The classification results demonstrated that compared with periodic-based EEG beta/theta ratios, the total MoCA total scores lead to better discrimination among clinical conditions. One potential reason is that performance on one cognitive assessment, e.g., the MoCA (or MMSE) is typically correlated with performance on other cognitive assessments such as the ones used to diagnose the clinical conditions. Nevertheless, the strong performance of the beta/theta ratio in distinguishing healthy individuals from those with AD and in correctly identifying AD among individuals with MCI vs. AD suggests that a 10-min resting state EEG could be complementary to clinical tools such as the MoCA or MMSE by providing more mechanistic information or when a clinical tool is not possible to be administered. Moreover, the high sensitivity of the beta/theta ratio in separating MCI from healthy individuals indicates that when an individual has a beta/theta ratio above the identified cut-off, one could be confident that the individual does not have MCI. This is particularly helpful when such an individual is scoring high on clinical screening cognitive tools due, for example, having high education yet the individual or their informant has cognitive concerns. Having a high beta/theta ratio on a resting-state EEG would further support the healthy status of such an individual.

We acknowledge the following limitations of this study. First, the sample size was insufficient to conduct subgroup analyses (e.g., based on sex). Second, these analyses were not conceived as part of the original studies. Third, the diagnoses of AD and MCI were based on clinical criteria without the use biomarkers. However, a clinical diagnosis based on the NINCDS-ADRDA criteria has been shown to be highly reliable [36, 37]. Thus, sample sizes may not have been large enough to detect significant differences and we needed to combine two data from two studies.

## Conclusions

Our findings support further study of the EEG periodic components based on beta/theta power ratio over the occipital lobe as a neurophysiological metric that separates patients with MCI and AD from healthy individuals. Future work to link this metric with other biological markers of neurodegenerative diseases are needed to further elucidate the mechanistic role of this metric.

### Supplementary Information

**Additional file 1:** CONSORT Charts for HC, MCI, and AD Participants.

## Data Availability

The data generated and analyzed in the current study is available from the corresponding author upon reasonable request.
