# Supplementary material for: Beta to theta power ratio in EEG periodic components as a potential biomarker in mild cognitive impairment and Alzheimer’s dementia
Source: Alzheimers Res Ther. 2023 Aug 7;15:133. doi: 10.1186/s13195-023-01280-z (PMC10405483; doi:10.1186/s13195-023-01280-z)
Supplement: Supplementary file 1 — Additional file 1: CONSORT Charts for HC, MCI, and AD Participants. [file 13195_2023_1280_MOESM1_ESM.docx]

**Supplementary Appendix**


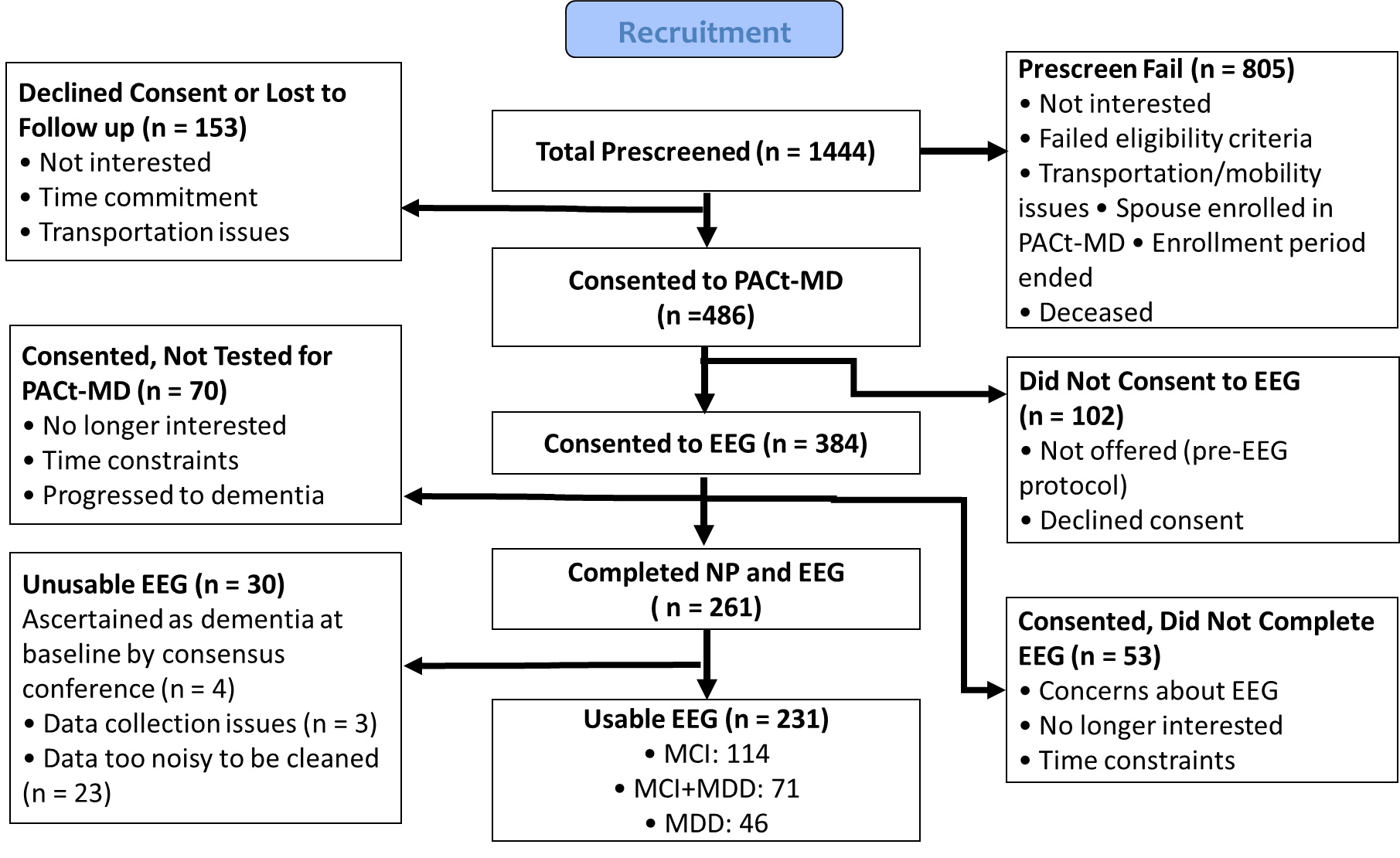


Figure S1. PACt-MD (Prevention of Alzheimer’s Dementia with Cognitive Remediation plus Transcranial Direct Current Stimulation in Mild Cognitive Impairment and Depression) CONSORT Chart for MCI Participants. EEG = Electroencephalography; MCI = Mild Cognitive Impairment; MDD = Major Depressive Disorder; NP = Neuropsychological Assessment.


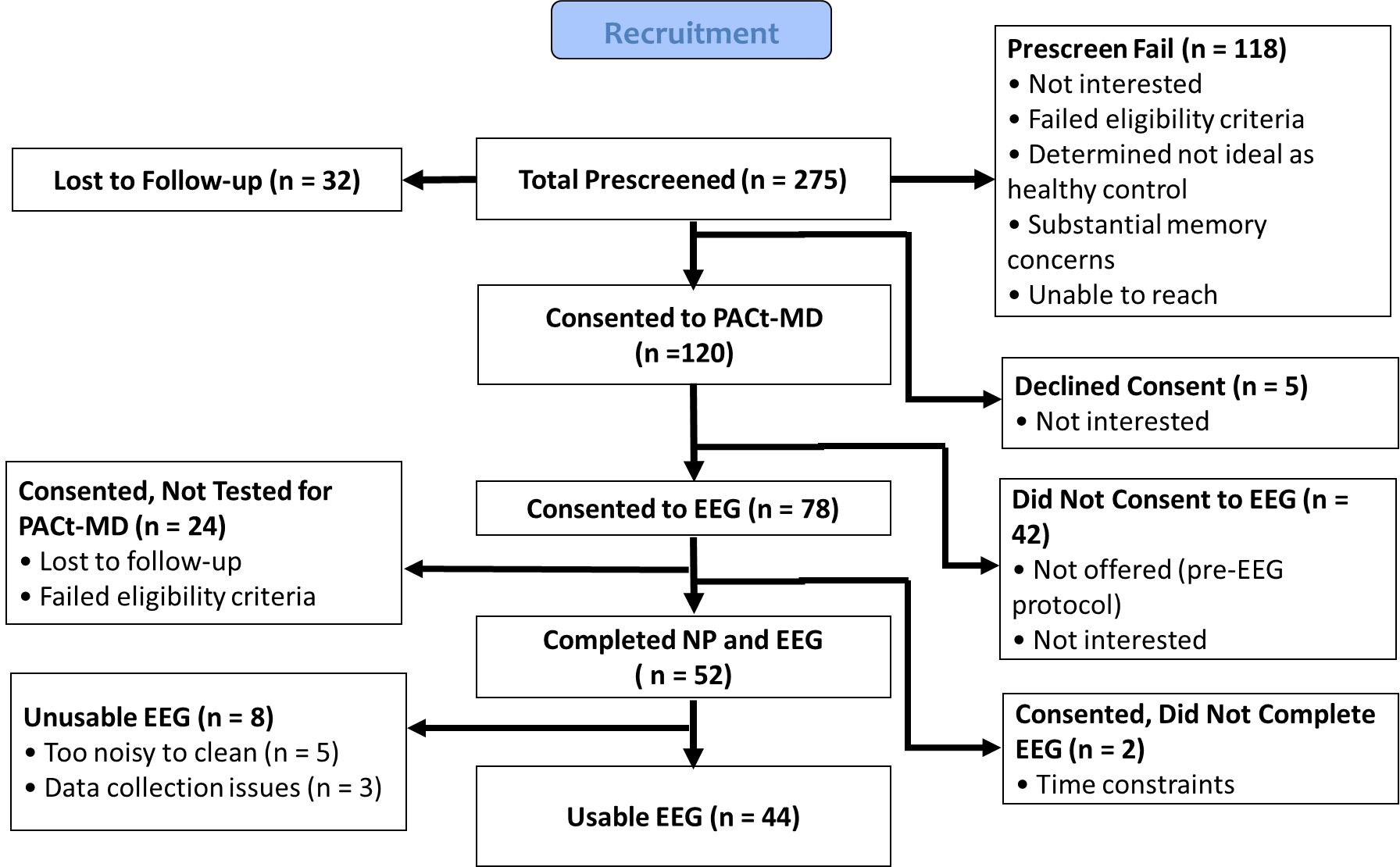


Figure S2. PACt-MD (Prevention of Alzheimer’s Dementia with Cognitive Remediation plus Transcranial Direct Current Stimulation in Mild Cognitive Impairment and Depression) CONSORT Chart for HC. EEG = Electroencephalography; HC = Healthy Control; NP = Neuropsychological Assessment.


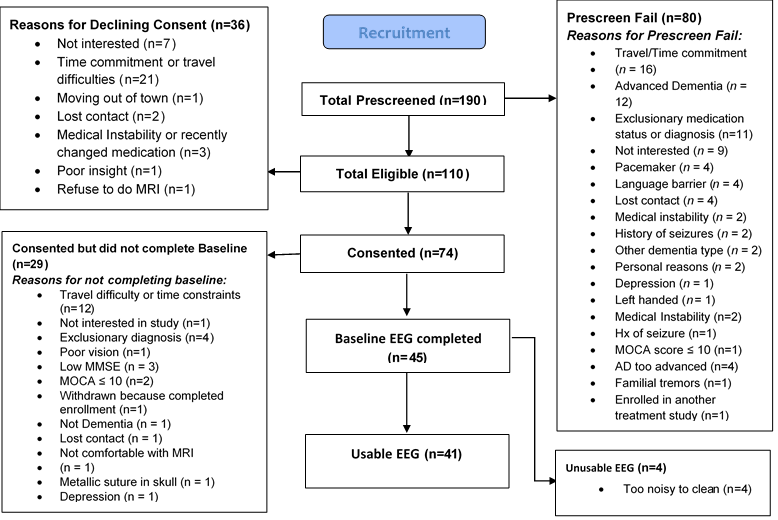


Figure S3. Alzheimer’s disease CONSORT Chart. EEG = Electroencephalography.
